# Gasdermin D silencing alleviates airway inflammation and remodeling in an ovalbumin-induced asthmatic mouse model

**DOI:** 10.1038/s41419-024-06777-5

**Published:** 2024-06-07

**Authors:** Jinxiang Wu, Pin Wang, Xinyu Xie, Xiaoqi Yang, Shuangmei Tang, Jiping Zhao, Tian Liu, Junfei Wang, Jintao Zhang, Tongliang Xia, Xin Feng

**Affiliations:** 1 Department of Pulmonary and Critical Care Medicine, Qilu Hospital, Shandong University, National Health Commission Key Laboratory of Otorhinolaryngology, Shandong University, Jinan, China; 2 Department of Otorhinolaryngology, Qilu Hospital of Shandong University, National Health Commission Key Laboratory of Otorhinolaryngology, Shandong University, Jinan, China; 3 Department of Respiratory, Shandong Qianfoshan Hospital, Cheeloo College of Medicine, Shandong University, Jinan, China

**Keywords:** Asthma, Chronic inflammation

## Abstract

Emerging evidence demonstrates that pyroptosis has been implicated in the pathogenesis of asthma. Gasdermin D (GSDMD) is the pyroptosis executioner. The mechanism of GSDMD in asthma remains unclear. The aim of this study was to elucidate the potential role of GSDMD in asthmatic airway inflammation and remodeling. Immunofluorescence staining was conducted on airway epithelial tissues obtained from both asthma patients and healthy controls (HCs) to evaluate the expression level of N-GSDMD. ELISA was used to measure concentrations of cytokines (IL-1β, IL-18, IL-17A, and IL-10) in serum samples collected from asthma patients and healthy individuals. We demonstrated that N-GSDMD, IL-18, and IL-1β were significantly increased in samples with mild asthma compared with those from the controls. Then, wild type and *Gsdmd*-knockout (*Gsdmd*^−/−^) mice were used to establish asthma model. We performed histopathological staining, ELISA, and flow cytometry to explore the function of GSDMD in allergic airway inflammation and tissue remodeling in vivo. We observed that the expression of N-GSDMD, IL-18, and IL-1β was enhanced in OVA-induced asthma mouse model. *Gsdmd* knockout resulted in attenuated IL-18, and IL-1β production in both bronchoalveolar lavage fluid (BALF) and lung tissue in asthmatic mice. In addition, *Gsdmd*^−/−^ mice exhibit a significant reduction in airway inflammation and remodeling, which might be associated with reduced Th17 inflammatory response and M2 polarization of macrophages. Further, we found that GSDMD knockout may improve asthmatic airway inflammation and remodeling through regulating macrophage adhesion, migration, and macrophage M2 polarization by targeting Notch signaling pathway. These findings demonstrate that GSDMD deficiency profoundly alleviates allergic inflammation and tissue remodeling. Therefore, GSDMD may serve as a potential therapeutic target against asthma.

## Introduction

Asthma is defined as a heterogeneous airway disease characterized by the key features of chronic inflammation along with airway tissue remodeling [1, 2]. However, the mechanisms underlying the regulation of inflammation along with airway remodeling are not fully understood.

Pyroptosis is a special programmed cell death mechanism that can induce strong inflammatory responses and proinflammatory cytokine release [3, 4]. Gasdermins (GSDMs), consisting of 6 members: gasdermins A-E and pejvakin, are central to the inflammatory process of pyroptosis and play various roles in pyroptosis [5]. Endogenous and exogenous danger factors activate caspase-1/4/5/11, which cleave gasdermin D (GSDMD) to its active isoform N-GSDMD. N-GSDMD then forms pores in the cell membrane, causing cell swelling and ultimate cell lysis. In the same process that caspases cleave GSDMD, they also cleave pro-IL-1β and pro-IL-18, converting them to their active forms. These cytokines are then released from the cell through the pores formed by N-GSDMD [3, 4]. Studies in recent years have found that pyroptosis contributes profoundly in the pathogenesis of asthma. House dust mites (HDM) increase the pyroptosis of airway epithelial cells and the release of inflammatory cytokines by activating the NOD-like receptor family pyrin domain containing 3 (NLRP3) inflammasome [6]. Furthermore, Gasdermin B (GSDMB) is highly expressed in the epithelial cells of ciliated airway, and multiple coding variants of GSDMB are associated with reduced risk of asthma [7, 8]. Fan liu et, al found that ORMDL3 may regulate pyroptosis and subsequent airway remodeling in obesity-associated asthma via the CTSD/NLRPP3/GSDMD pathway [9]. Highly expressed miR-223-3p in exosomes may protect against airway remodeling and asthma by regulating the NLRP3-induced ASC/Caspase-1/GSDMD signaling pathway [10]. However, the function of GSDMD in allergic or other subtypes of asthma and the underlying mechanism require further exploration.

Asthma has been widely acknowledged as a T helper 2(Th2) cell and T helper 17 (Th17) cell-mediated inflammatory disease that contributes to airway hyperresponsiveness (AHR) and airway inflammation [11–14]. Notably, macrophages are the most abundant immune cells in the lung (approximately 70% of the immune cells are macrophages) [15]. Increased M2 polarization and activation of macrophages may play an important role in environmental airway inflammation in allergic asthma [16, 17]. However, understanding of the molecular mechanisms regulating macrophage polarization and Th17 responses in asthma is limited. Here, we detected the expression level of gasdermin D in the airway epithelium of asthma patients. We then used wild-type (WT) and Gsdmd-knockout mice to establish an asthma mouse model. We investigated the role of GSDMD in the pathogenesis of asthma. Overall, we investigated the function and underlying mechanism of GSDMD in asthmatic airway inflammation and remodeling.

## Materials and methods

### Patients and clinical characteristics

Epithelium tissues from asthmatic patients (n = 7) were obtained by bronchoscopy at Qilu Hospital, Shandong University (Jinan, Shandong, China). The control specimens (n = 7) were obtained from the normal tissue adjacent to tumor of lung cancer during thoracic surgery at Qilu Hospital.

Serum specimens were obtained from peripheral blood samples of 20 asthmatic patients and 20 healthy individuals from Qilu Hospital. The diagnosis of asthma was accomplished according to the Global Initiative for Asthma (2020 updated) [18]. Control subjects did not have asthma according to their history. Exclusion criteria were current smoking, severe comorbidities including bronchiectasis, allergic bronchopulmonary aspergillosis (ABPA), and respiratory tract infection or acute exacerbation within the previous 4 weeks. This study was approved by the Medical Ethics Committee of Qilu Hospital of Shandong University. Consent was obtained from all subjects. The clinical characteristics are shown in Table 1.

**Table 1:** Epidemiological and clinical characteristics of the participants used for the cytokines evaluation.

Characteristic	Control (n = 20)	Asthma (n = 20)	*P* value
Age (years)^a^	45.2 ± 3.3	49.05 ± 9.8	0.105
Sex^b^			0.327
Female	9	6	
Male	11	14	
BMI (kg/m^2^)^a^	25.3 ± 2.9	24.1 ± 3.3	0.253
FEV1 (L)^a^	3.3 ± 0.7	1.9 ± 0.6	0.000
FEV1 (% of predicted)^a^	109.4 ± 14.7	64.3 ± 11.8	0.000
FEV1/FVC (% of predicted)^a^	98.3 ± 5.6	73.7 ± 1.0	0.000
EOS count (*10^9^)^a^	0.08 ± 0.04	0.31 ± 0.23	0.000
EOS percentage (%)^a^	1.5 ± 1.1	5.1 ± 4.4	0.000

Values are means ± SD or as noted.

*BMI* body mass index, *FEV1* forced expiratory volume in 1 s, *FEV1%* FEV1 percentage of predicted, *FVC* forced vital capacity, *EO*S count eosinophilic count, *EOS%* eosinophilic percentage of leukocyte.

^a^Two-sample t-test;

^b^Chi-square test.

### Animals

Wild-type female C57BL/6J mice aged 6–8 weeks were purchased from SPF (Beijing) Biotechnology Co., Ltd., and raised in specific pathogen-free facility prior to experiments. *Gsdmd* knockout mice (*Gsdmd*^*−/−*^) were generous gifts from Dr. Feng Shao (Investigator and Deputy Director for Academic Affairs, NIBS, Beijing, China) and maintained in the Animal Facility at Shandong University. No blinding was done in animal study. The protocols for animal experiments were reviewed and approved by the Institutional Animal Care and Use Committee of Qilu Hospital of Shandong University.

### Differentiation and stimulation of macrophages

Bone marrow-derived macrophages (BMDMs) were differentiated from the bone marrow cells of 6–8 week old female C57BL/6 J mice according to the published procedures [19]. Briefly, bone marrow cells were isolated from the femurs and tibias and cultured in RPMI 1640 medium supplemented with 10% FBS, 50 μg/ml Gentamicin and 20 ng/ml macrophage colony-stimulating factor (M-CSF). Cells were differentiated for 6 days [19]. Macrophages, seeded at a density of 1–5 × 10^6^ cells per well in 6-well plates, were incubated for 24 h either in medium alone or treated with recombinant TSLP (10 ng/ml) [20] for different assays.

### Enzyme-linked immunosorbent assay (ELISA)

The concentrations of IL-18 (ab215539, abcam), IL-1β (ab217608, abcam), IL-17A (ab216167, abcam) and IL-10 (ab185986, abcam) in human peripheral blood serum, and IL-4 (ab100710, abcam), IL-5 (ab204523, abcam), IL-18 (ab216165, abcam), IL-1β (ab241673, abcam), IL-17A (ab199081, abcam) and IL-10 (ab255729, abcam) in mouse BALF were determined by ELISA.

### Analysis of bronchoalveolar lavage fluid (BALF)

BALF from all groups was collected in 1 ml of PBS. The collected lavage fluid was centrifuged at 500 × *g* for 5 min at 4 °C. The precipitated cells were resuspended in 0.2 mL of PBS. BALF cell smears were prepared using cytospin slides. The slides were stained using Wright-Giemsa staining (Fisher Scientific Co., Middletown, VA). The cell counts and cytokines in BALF were further determined by two certified laboratory technicians who were blinded to the experimental conditions.

### Establishment of the allergic asthma mouse model

All mice were randomly divided into groups and sensitized by intraperitoneal (i.p.) injection of 50ug ovalbumin (OVA; grade V, Sigma, St Louis, MO, USA) and 2 mg aluminum hydroxide (Thermo Scientific Pierce, Rockford, Rockford, IL, USA) in 200 μl phosphate buffered saline (PBS) on days 0 and 14. From days 21 to 27, the sensitized mice were exposed to aerosolized OVA (5% in PBS) for 30 min daily. Non-OVA challenged mice including WT and *Gsdmd−/−* control groups mice were sensitized and challenged with PBS only. All the mice were sacrificed 24 h after the last challenge. Serum, BALF, lung, and spleen tissues were collected for further experiments. The BALF was used for cytokine detection by ELISA. The lung tissues were used for hematoxylin and eosin (H&E), immunofluorescence staining, immunohistochemistry, Periodic acid-Schiff (PAS) staining, Sirus staining, Masson trichrome staining, transmission electron microscopy, quantitative real-time PCR, and Western blot analysis. Spleen tissues were used for analysis of inflammatory cells by flow cytometry. A semi-quantitative scoring system was used for quantification of tissue histopathology [21].

### Transmission electron microscopy (TEM)

Human and mouse lung tissues were fixed and prepared as previously described [22]. Briefly, the ultrathin lung sections were cut with an ultracut microtome (Leica, Solms, Germany), and were stained with 4% uranyl acetate and lead citrate. The images were then observed and photographed using a JEM-100cxII transmission electron microscope (JEM, Tokyo, Japan).

### Immunofluorescence staining and immunohistochemistry

Immunohistochemistry and immunofluorescence staining were performed as described previously [22–24]. Briefly, slides were incubated with anti-GSDMD reacted with mouse (ab209845, abcam) (1:50), anti-GSDMD reacted with human (ab155233, abcam) (1:50), anti-IL-18 (ab243091, abcam) (1:200), anti-IL-1β (ab254360, abcam) (1:200), anti-CD206 (ab64694, abcam) (1:200), anti-NOS2 (sc7271, Santa Cruz) (1:200), and anti-F4/80 (RT1212, HUABIO) (1:200) antibodies for immunofluorescence staining, respectively. The expression of α-SMA and collagen I was assessed by immunohistochemistry using anti-α-smooth muscle actin (α-SMA) antibody (1:200; Boster, Wuhan, China), and anti-collagen type I (1:100; Proteintech, Wuhan, China) antibodies. The slides were then processed with the appropriate secondary antibodies/horseradish peroxidase (1:200).

### Histological analysis

Mouse lung tissues were fixed in 10% neutral formalin, paraffin embedded, and sectioned at 4 μm. After deparaffinization, the sections were rehydrated and used for histological analysis. Inflammation scores in the lungs were determined by HE staining and graded according to a previous study [25]. The mean percentage of collagen deposition was calculated by Sirius and Masson trichrome staining, and quantified as described in the research [26]. Goblet cell hyperplasia was evaluated by PAS staining as described by Padrid et al. [27].

### Western blot analysis

Western blot was performed using standard protocol as previously described [28]. The following primary antibodies were used: anti-GSDMD (ab209845, abcam), anti-IL-18 (ab243091, abcam) (1:2000), anti-IL-1β (ab254360, abcam) (1:2000), anti-caspase-1 (ab180673, abcam) (1:1000), anti-caspase-11(ab138483, abcam) (1:1000), anti-Notch 4 (A-12) (sc-393893, Santa Cruz) (1:1000), anti-DTX4 (25222-1-AP, Proteintech, 1:100), and anti-β-actin (ab8226, abcam) (1:10000) antibodies. β-actin was used as an internal control.

### Determination of AHR

Airway function was measured as previously described [29]. The results were expressed as the maximal resistance after each dose minus the baseline resistance (PBS alone). Data were analyzed using Datanalyst software (DATA 4238).

### Flow cytometry analysis

For intracellular cytokine staining, the fresh isolated splenocytes from C57BL/6 and *Gsdmd*^*−/−*^ mice were stimulated with PMA (50 ng/ml) and Ionomycin (1 µg/ml), and incubated in complete RPMI 1640 medium at 37 °C for 3 h. Then, brefeldin A was added and cells were cultured for another 3 h to accumulate cytokines intracellularly. Cells were collected and blocked with purified anti-CD16/CD32 in FACS staining buffer for 10 min, and surface stained with anti-CD3-PerCP-Cy5.5 (11-0161-82, ebioscience, USA), anti-CD4-FITC mAb (ebioscience, USA). Then, cells were fixed, permeabilized and intracellularly stained with anti-IL-17A-PE (12-7177-81, invitrogen) for 30 min.

To detect Treg, after surface stained with anti-CD3-FITC (11-0031-85, ebioscience), anti-CD4-APC (17-0041-81, ebioscience) and anti-CD25-PE mAb (12-0251-81, invitrogen), cells were fixed with Fixation/Permeabilization buffer (00-5223-56, eBioscience) for 30 min in 4 °C. Then, cells were washed with Permeabilization buffer, and stained with anti-Foxp3- PerCP-Cy5.5 antibody (45-5773-82, ebioscience) in Permeabilization buffer for 30 min at room temperature. Antibody-labeled cells were analyzed using BD FACS Calibur (BD Biosciences, San Jose, CA).

BMDMs from WT and *Gsdmd*^*−/−*^ mice were induced for 6 days and then stimulated with TSLP (10 ng/ml) or PBS. Cells were collected and blocked for 10 min with anti-CD16/CD32 in FACS staining buffer. Then, cells were stained with anti-CD11b-FITC (11-0112-82, invitrogen), anti-F4/80-APC (11-4801-82, invitrogen), anti-CD206-PE-cy7 (25-2061-80, ebioscience, USA), anti-CD86-PE mAb (12-0861-81, ebioscience, USA) for 30 min in 4 °C. Antibody-labeled cells were detected using BD FACS Celesta. All the data of flow cytometry analysis were subsequently analyzed with FlowJo 10.4.

### RNA extraction and quantitative real-time PCR

Total RNA was extracted from lung tissues and macrophages of WT mice and *Gsdmd*^*−/−*^ mice by TRI reagent (Cat. No. T9424; Sigma-Aldrich; Merck KGaA) following the manufacturer’s instructions. Reverse transcription was performed using the HiScript III RT SuperMix for qPCR (+gDNA wiper) (fCat. No. R323-01; Vazyme Biotech Co., Ltd.). Reverse transcription quantitative polymerase chain reaction (RT-qPCR) was performed as previously described [30]. cDNA was synthesized from 1 μg of total RNA using HiScript III RT SuperMix (cat. no. R323-01; Vazyme Biotech Co., Ltd.). qPCR was performed using a LightCycler480II Real-time PCR system (Roche Diagnostics (Shanghai) Co., Ltd, Shanghai, China) with SYBR^®^ Green-based gene expression analysis. A comparative CT method (2^-ΔΔCq^) was used to analyze the gene expression level as previously described [31]. The primers targeting genes of Notch signaling were listed in Table 2.

**Table 2:** PCR primers for target genes.

Primers	Sequence (5′ > 3′)
Efnb2-F	5′-ATTATTTGCCCCAAAGTGGACTC-3′
Efnb2-R	5′-GCAGCGGGGTATTCTCCTTC-3′
Notch4-F	5′-CTCTTGCCACTCAATTTCCCT-3′
Notch4-R	5′-TTGCAGAGTTGGGTATCCCTG-3′
Acvrl1-F	5′-GGGCCTTTTGATGCTGTCG-3′
Acvrl1-R	5′-TGGCAGAATGGTCTCTTGCAG-3′
Lrrc32-F1	5′-TCAGCGTCGAGAGCAAGTG-3′
Lrrc32-R1	5′-GTAGAGAGCTTGGATGTCCAGT-3′
Bcam-F	5′-TCAGCGTCGGTCTTTTGCTAC-3′
Bcam-R	5′-CAACACTCATCTCCAAAGCCTC-3′
Dtx4-F	5′-TGTGCCTGTGAAAAACTTGAATG-3′
Dtx4-R	5′-TGGGATGGACTTTATCTCACTCT-3′
Hes1-F	5′-CCAGCCAGTGTCAACACGA-3′
Hes1-R	5′-AATGCCGGGAGCTATCTTTCT-3′

### Bulk RNA sequencing (RNA-seq) and data processing

Bone marrow-derived macrophages were lysed in TRIzol reagent. Subsequent total RNA extraction, cDNA library preparation and RNA sequencing were carried out by Novogene Co, Ltd. Differentially expressed genes (DEGs) analysis was performed on the sequencing results using the ‘limma’ R package. Functional annotation, Gene Ontology (GO) enrichment, and Kyoto Encyclopedia of Genes and Genomes (KEGG) enrichment analysis were performed utilizing the“ClusterProfiler” package to predict the biological function and related pathways. The top six enriched terms in the biological process (BP), cellular component (CC), and molecular function (MF) were visualized using the “ggplot2” and “enrichplot” R packages. Reactome pathway analysis was performed using the “ReactomePA” R package. The top ten enriched terms were presented. *P* value < 0.01 were selected for presentation. Gene set enrichment analysis (GSEA) was conducted using GSEA v4.3.3 software with hallmark gene sets. The RNA-seq data were deposited with the Gene Expression Omnibus (GEO) under accession number GSE 249948.

### Statistical analysis

The data generated in this study were analyzed using Graph Pad Prism 8 and SPSS version 26.0. Continuous data were presented as mean with standard deviation (SD) or standard error of the mean (SEM), and were assessed for normality and equal variation. The chi-square test was applied for categorical variables to compare the demographic distribution and clinical variables between the different groups. One-way analysis of variance (ANOVA) was performed for comparisons of multiple groups that passed the normality and equal variation tests, otherwise, Kruskal–Wallis test was used. Subsequent multiple comparisons between two groups were further adjusted using the Holm–Sidak’s multiple comparisons test and Dunn’s multiple comparisons test, respectively. Student’s t was performed between two groups if the data were normally distributed, otherwise it was assessed by Mann-Whitney test (*P* < 0.05 was considered statistically significant).

## Results

### GSDMD and pyroptosis are highly involved in asthma pathology

**Fig. 1:**
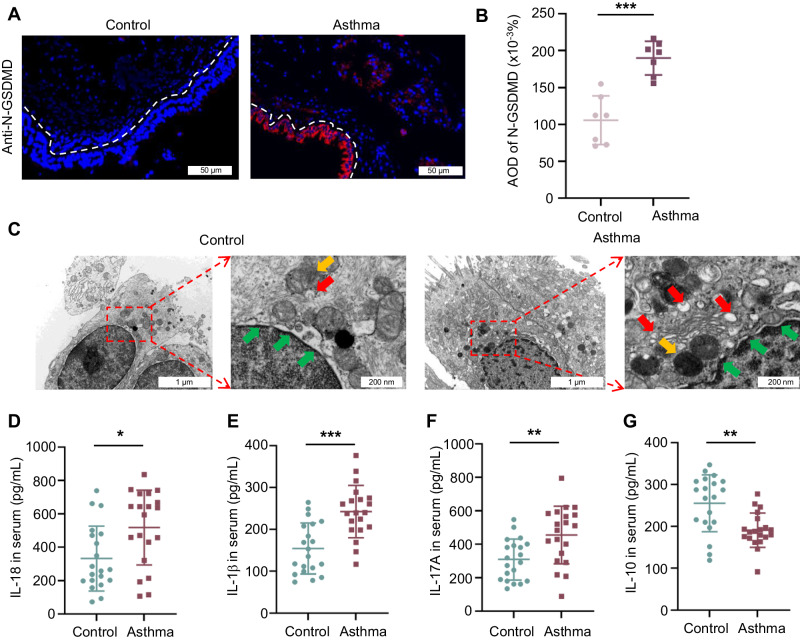
Clinical implications of GSDMD-mediated pyroptosis in asthma. **A** Immunofluorescence staining of N-GSDMD in human airways. **B** Average optical density (AOD) of N-GSDMD in airway epithelium between control and asthma patients (n = 7 per group). **C** Ultrathin lung sections were scanned using TEM to assess pyroptosis of airway epithelium in asthma patients and the control group. The red arrow indicates the pyroptosis-related vacuolization structures, the yellow arrow indicates the mitochondria, the green arrow indicates the nuclear membrane of the nucleus. Scale bars: 1μm and 200 nm as indicated. Expression of serum (**D**) IL-18, (**E**) IL-1β, (**F**) IL-17A, and (**G**) IL-10 were detected in asthma patients and in the control group (n = 20 per group). All the data are presented as means ± SD. *P < 0.05, **P < 0.01, ***P < 0.001.

N-GSDMD acts as the final and direct executor of pyroptosis. To investigate whether the pyroptosis is involved in asthma, we first evaluated the protein expression of the pyroptosis-inducing fragment N-GSDMD in lung biopsies from 7 asthma patients and 7 controls. Analysis of immunofluorescence staining and HE staining showed that N-GSDMD was highly expressed in the airway epithelium of asthma patients compared to the control group (Fig. 1A, B, Supplementary Fig. 1A). Ultrathin lung sections were scanned by TEM to assess the pyroptosis of the airway epithelium. Some pyroptotic morphological features, such as cytoplasmic swelling, bubbling, and nuclear condensation were detected in the ultrathin sections of the airway epithelium from asthma patients but not from controls (Fig. 1C). The production of pyroptosis-associated inflammatory cytokines IL-18 and IL-1β was higher in the serum of asthma patients compared to that in the controls (Fig. 1D, E). To detect the imbalanced Th17/Treg in asthma, we examined the release of Th17 cell-associated IL-17A and Treg cell-associated IL-10. The secretion of IL-17A was also increased in the serum from the asthmatic group. In contrast, IL-10 production was decreased in the serum from the asthmatic group (Fig. 1F, G). These data suggested that pyroptosis is enhanced in asthma.

### *Gsdmd* deficiency alleviates pyroptosis in the OVA-induced asthma mouse model

**Fig. 2:**
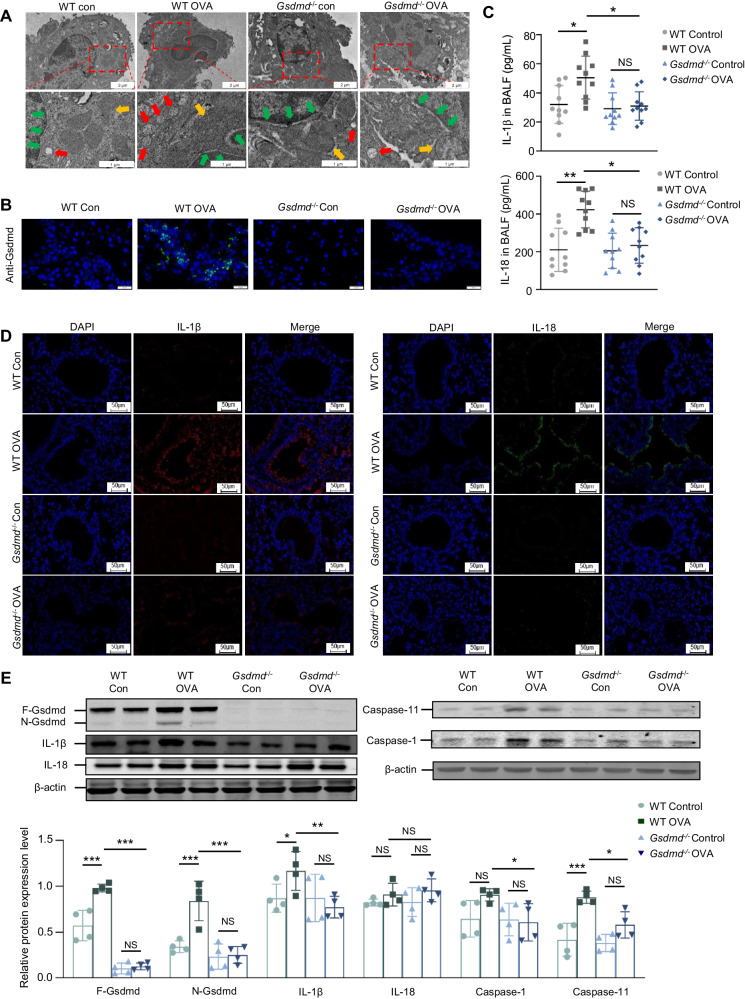
*Gsdmd* deletion attenuates pyroptosis in asthmatic mice. **A** Ultrathin lung sections were scanned using TEM to assess the pyroptosis of airway epithelium cells. The red arrow indicates the pyroptosis-related vacuolization structures, the yellow arrow indicates the mitochondria, the green arrow indicates the nuclear membrane. Scale bars: 2μm and 1μm as indicated. **B** The expression of Gsdmd detected by immunofluorescence staining in the lung tissue. Scale bars: 20 μm. **C** The secretion of IL-1β and IL-18 in the BALF of mice from the indicated group; n = 10 per group. **D** Representative images of lung sections from WT and *Gsdmd*^*−/−*^ mice immunostained with anti-IL-18 and anti-IL-1β antibodies. Scale bars: 50 µm; n = 5 per group. **E** The protein level and quantification of F-Gsdmd, N-Gsdmd, IL-18, IL-1β, Caspase-1, and Caspase-11 were examined in the lungs of WT and *Gsdmd*^*−/−*^ *mice*. β-actin is the internal control. n = 5 per group. No significance is indicated as NS. Results are presented as mean ± SD; *P < 0.05, **P < 0.01, ***P < 0.001.

To further characterize the role of Gsdmd in regulating pyroptosis in asthma, an OVA-induced asthma mouse model was established. Mice were randomly divided into four groups, including PBS-treated wild type mice (WT control), OVA-treated wild type mice (WT OVA), PBS-treated *Gsdmd* knockout mice (*Gsdmd*^*−/−*^ control), and OVA-treated *Gsdmd* knockout mice (*Gsdmd*^*−/−*^ OVA) (Supplementary Fig. S1B). Morphological changes in the airway epithelium of WT and *Gsdmd*^*−/−*^ asthmatic mice were examined by TEM. As predicted, a number of pyroptotic morphological features, such as cytoplasmic swelling, bubbling, osmotic lysis, and nuclear condensation, were widely observed in the airway epithelium of WT OVA mice, while the pyroptosis of the airway epithelial cells was mitigated in the *Gsdmd*^*−/−*^ OVA mice (Fig. 2A). To assess the expression of Gsdmd in the asthma model, immunostaining with anti-Gsdmd antibody was performed on the lung tissue sections. Gsdmd was significantly upregulated in WT OVA mice compared with those from WT control mice (Fig. 2B). The secretion of IL-18 and IL-1β, two pyroptosis-associated inflammatory cytokines, was increased in the BALF from the WT OVA mice, while no significant change in IL-18 and IL-1β production was detected in the *Gsdmd*^*−/−*^ OVA mice (Fig. 2C). The effect of Gsdmd on IL-18 and IL-1β expression was further validated by immunofluorescence staining (Fig. 2D) and WB analysis (Fig. 2E) on the lung tissues of asthmatic mice. IL-18 did not show significant difference among groups in WB detection, which may be ascribed to different mechanisms through which IL-1β and IL-18 are expressed in cells and individual heterogeneity within groups. Caspase-1 and caspase-11 are regarded as the hallmark proteins of cell death in the pyroptosis progress [4]. Here we observed remarkable increase of caspase-11 and caspase-1 in lung tissues from OVA-induced asthmatic mice, which further addressed the pyroptosis in asthmatic mice. In contrast, only mild changes in caspase-1 and caspase-11 expression were detected in the *Gsdmd*^*−/−*^ OVA mice as compared to the *Gsdmd*^*−/−*^ control mice (Fig. 2E). Thus, *Gsdmd* deficiency may attenuate pyroptosis in asthma.

### *Gsdmd* deficiency attenuates AHR and airway inflammation in the OVA-induced asthma mouse model

**Fig. 3:**
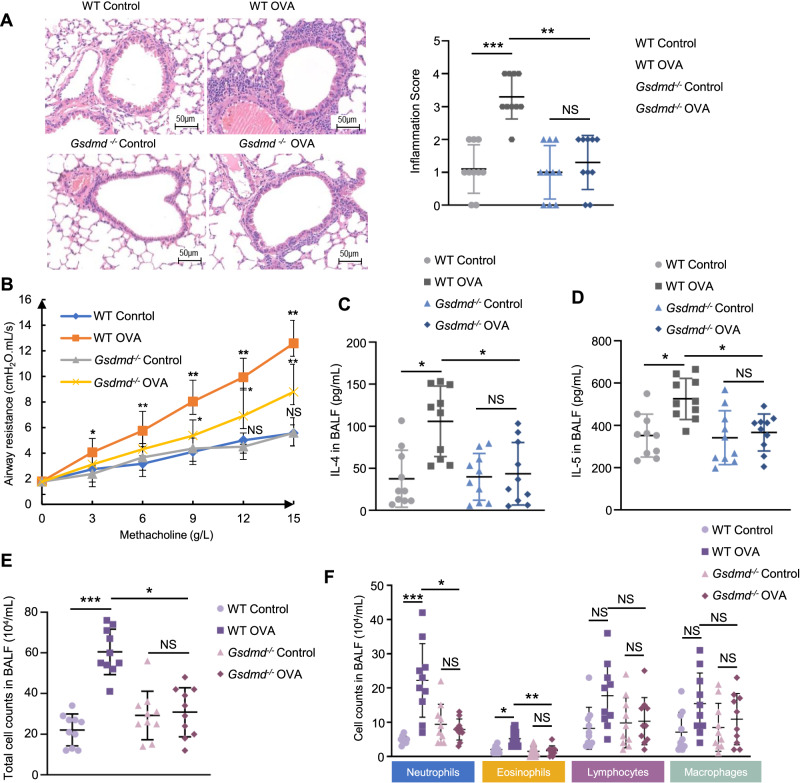
*Gsdmd* deficiency reduces the AHR and type 2 immune responses in OVA-induced asthmatic mice. **A** The morphological changes of airway were observed by HE staining (left panel). The inflammatory infiltration was quantified by HE scores (right panel). Scale bars: 50 µm. **B** AHR to increasing doses of methacholine was measured 24 h after the last challenge on WT and *Gsdmd*^*−/−*^ mice by recording changes in lung resistance. **C** IL-4 and **D** IL-5 secretion in mouse BALF were detected by ELISA. **E** Total cells number in BALF were counted. Mice groups were the same with panel c and d. **F** The numbers of neutrophils, eosinophils, lymphocytes and macrophages in the mouse BALF were detected. Results are expressed as mean ± SD. No significance is indicated as NS. n = 10 per group for all experiments. *P < 0.05, **P < 0.01, ***P < 0.001.

Asthma is characterized by chronic airway inflammation leading to increased AHR. HE staining showed that *Gsdmd* knockout significantly alleviated the airway inflammation by reducing the inflammatory cell infiltration (Fig. 3A). To further explore the pathogenic effect of Gsdmd on asthmatic airway inflammation and AHR, we examined airway resistance and production of airway inflammation-related cytokines in both WT and *Gsdmd*^−/−^ asthmatic mice. An evident increase in AHR was detected in WT OVA mice as compared to *Gsdmd*^−/−^ OVA mice, suggesting a protective role of *Gsdmd* silencing in the OVA-induced asthma (Fig. 3B). In addition, the secretion of IL-4 and IL-5 was decreased in the BALF of *Gsdmd*^−/−^ mice (Fig. 3C, D). Meanwhile, the count of total cells, neutrophils, and eosinophils in BALF were markedly decreased by *Gsdmd* knockout in asthmatic mice (Fig. 3E, F). These results imply that silencing *Gsdmd* may serve as a therapeutic strategy against asthma by reducing AHR and airway inflammation.

### *Gsdmd* deficiency suppresses airway remodeling in the OVA-induced asthma mouse model

**Fig. 4:**
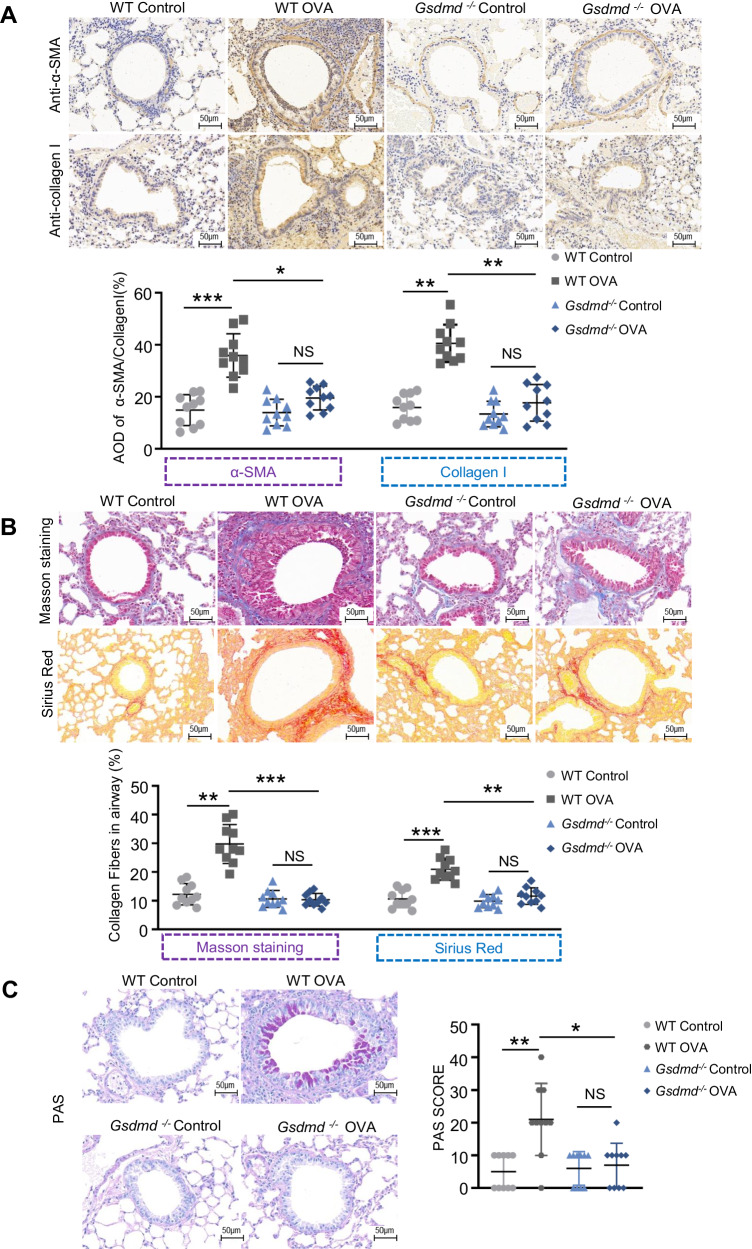
*Gsdmd* deficiency ameliorates airway remodeling in asthmatic mice. **A** Immunohistochemistry staining for α-SMA and collagen I in lung tissue of mice (Scale bar: 50 μm) (upper panel), and the expression of collagen I and α-SMA were quantified (lower panel). **B** Representative images of Masson’s staining measuring smooth muscle hyperplasia and Sirius red measuring collagen deposition (Scale bar: 50μm) (upper panel). The collagen deposition was quantified (lower panel). **C** Representative images of PAS-stained lung sections from each group (Scale bars: 50 µm) (left panel). The goblet cell hyperplasia was determined by PAS scores (right panel). The data are expressed as mean ± SD. No significance is indicated as NS. n = 10 per group for all experiments. *P < 0.05, **P < 0.01, ***P < 0.001.

Next, we investigated the role of Gsdmd in airway remodeling. Asthmatic airway remodeling is mainly associated with collagen deposition, mucus overproduction, and high expression of remodeling markers. Consistent with our previous findings [22], we found that the protein levels of the remodeling markers, α-SMA and collagen I, were significantly increased in the airway epithelium of the asthma model (Fig. 4A). Collagen fiber content was dramatically increased in airway epithelium of the asthma model as compared to the control group, as shown by Masson’s trichrome staining and Sirius red staining (Fig. 4B). PAS staining showed that the OVA-challenged mice had obvious mucus production in the epithelial cells (Fig. 4C). Compared to WT asthmatic groups, OVA challenge induced increased levels of remodeling markers, collagen deposition and mucus overproduction, which were attenuated by *Gsdmd* knockout in asthmatic mice (Fig. 4A–C). These data suggest that *Gsdmd* deficiency is effective in preventing airway remodeling in OVA-induced asthma.

### *Gsdmd* plays important roles in asthma by impairing the Th17/Treg cell imbalance

**Fig. 5:**
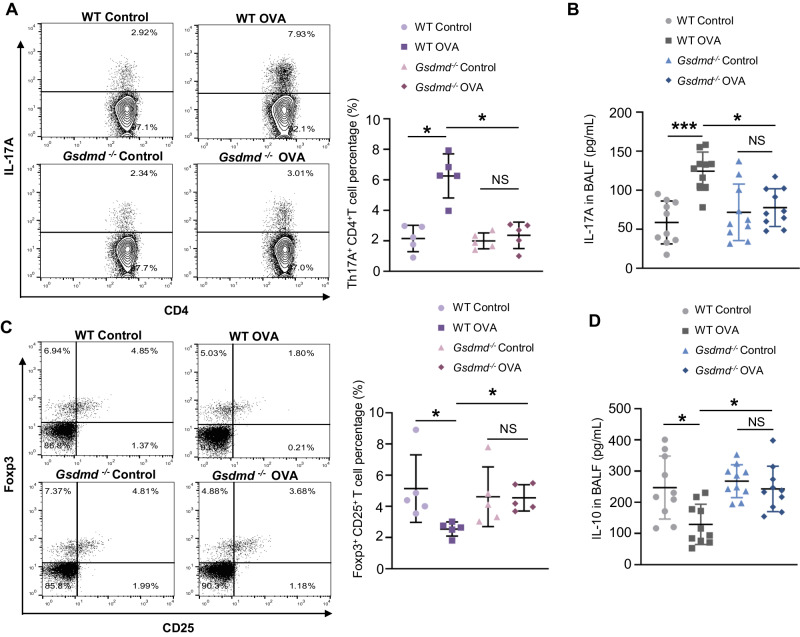
*Gsdmd* deficiency eliminates Th17/Treg imbalance in asthmatic mice. The spleen cells were labeled using indicated antibodies in “Method” section. The labeled cells were analyzed by flow cytometry. **A** Th17 cells were detected by flow cytometry, and the percentage of Th17 cells was quantified; n = 5 per group. **B** Secretion of IL-17A in mouse BALF was detected using ELISA; n = 10 per group. **C** Treg cells were analyzed by flow cytometry, and the percentage of Treg cells was quantified; n = 5 per group. **D** The secretion of IL-10 in mouse BALF was detected by ELISA; n = 10 per group. The data are presented as mean ± SD. Not significant is indicated as NS. *P < 0.05, ***P < 0.001.

The spleen is the largest immune organ, and the center of cellular and humoral immunity in the body. To investigate whether Gsdmd promotes asthma progression by altering the Th17/Treg balance, we collected the spleen tissue from mice in WT control, WT OVA, *Gsdmd*^*−/−*^ control, and *Gsdmd*^*−/−*^ OVA groups for flow cytometry analysis. We found that the percentage of Th17 (IL17A^+^CD4^+^CD3^+^) cells was dramatically increased in the spleen of the WT asthmatic mice compared to those from the control mice, whereas *Gsdmd* deficiency reversed the increase of Th17 cells upon OVA stimulation (Fig. 5A). Meanwhile, we detected the level of IL-17A associated with Th17 cells in BALF and obtained the consistent results (Fig. 5B). Correspondingly, the percentage of Treg (CD3^+^CD4^+^CD25^+^FOXP3^+^) cells in the spleen and IL-10 related to Treg cells were decreased in the WT asthmatic mice, whereas *Gsdmd* deficiency reversed the decrease of Treg cells (Fig. 5C) and IL-10 release (Fig. 5D). These findings suggested that the Th17/Treg imbalance, which is increased in asthma, is strongly associated with Gsdmd.

### *Gsdmd* deficiency mitigates macrophage infiltration in the OVA-induced asthma mouse model

**Fig. 6:**
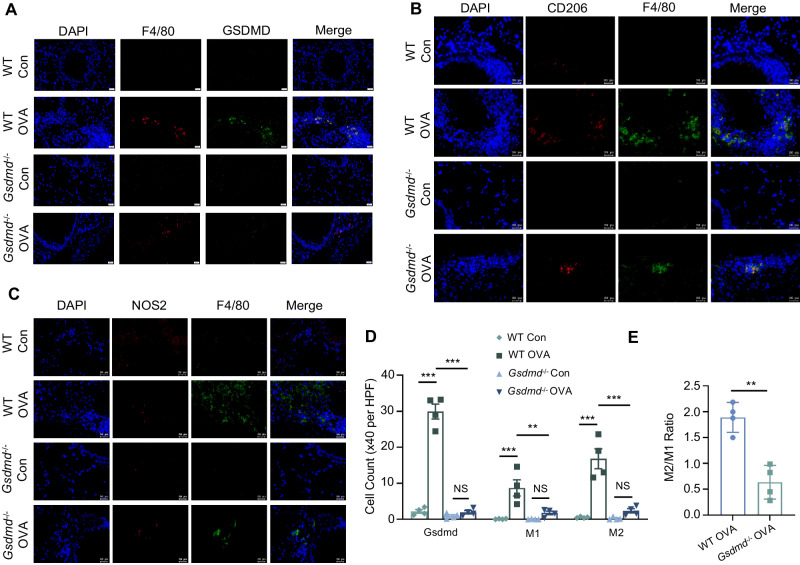
*Gsdmd* deficiency reduces macrophage infiltration in vivo. Representative images of immunofluorescence staining with antibodies targeting (**A**) F4/80 and GSDMD, (**B**) F4/80 and M2 macrophage maker CD206, (**C**) F4/80 and M1 macrophage maker NOS2 in the lung tissue. Scale bars: 20 µm. **D** The counts of macrophages and GSDMD positive cells were quantified; n = 4 per group. **E** The M2/M1 ratio of macrophages was only quantified between OVA-induced WT mice and OVA-induced Gsdmd^−/−^ mice, as the M2 and M1 ratio in WT mice and Gsdmd^−/−^ mice are too low to be calculated and be used for statistical analysis; n = 4 per group. The data are presented as mean ± SEM. Not significant is indicated as NS. *P < 0.05, **P < 0.01, ***P < 0.001.

It has been widely acknowledged that M2 macrophage polarization plays a significant role in inflammation and tissue remodeling of asthma over the last few decades [32]. To ascertain the function of GSDMD in regulating M1/M2 polarization in asthma, we firstly assessed the protein expression of Gsdmd in macrophages in the asthma model by immunostaining on the lung tissue. Gsdmd was significantly upregulated in F4/80 positive cells in lung tissue sections from asthmatic mice compared with those from control mice (Fig. 6A, D). As expected, the colocalization of Gsdmd, F4/80 was not apparent in lung tissue sections from two groups of Gsdmd knockout mice. Consistent with previous study, the number of M2 macrophages was increased in lung mucosa of asthmatic mice [33] (Fig. 6B, D). In addition, higher expression of the M1 macrophage marker NOS2 was also observed in lung tissue sections from asthmatic mice compared with control mice, as well as the enhanced macrophage infiltration (Fig. 6C, D). The macrophage M2/M1 ratio was significantly reduced in Gsdmd^−/−^ asthmatic mice compared to that in WT asthmatic mice, reflecting the attenuation of inflammation led by Gsdmd deficiency (Fig. 6E). In summary, Gsdmd deficiency alleviates macrophage infiltration in an asthmatic animal model.

### *Gsdmd* deficiency alleviates TSLP-induced M2 macrophage polarization

**Fig. 7:**
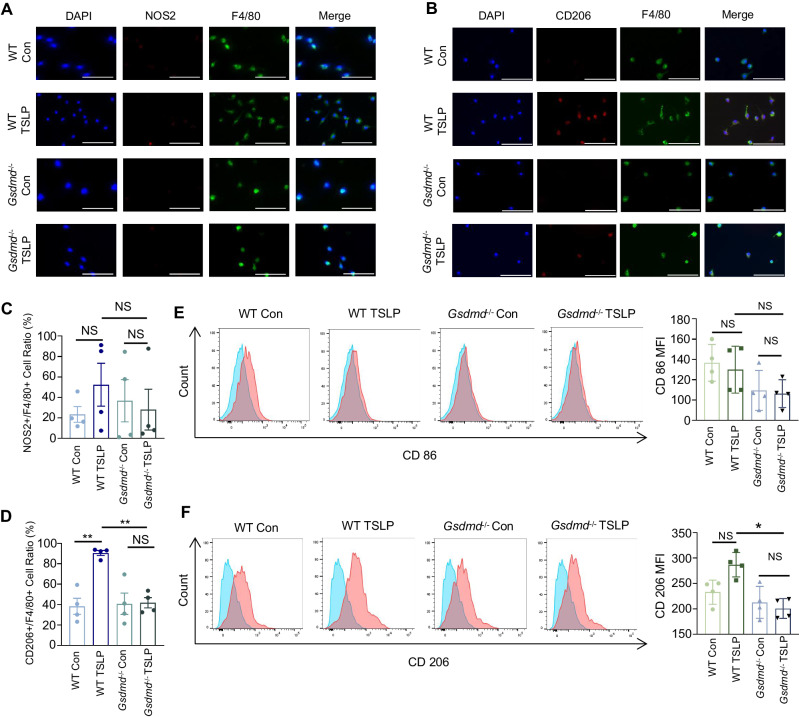
*Gsdmd* deficiency reduces macrophage polarization in vitro. Isolated bone marrow-derived macrophages obtained from WT and *Gsdmd*^*−/−*^ mouse models were treated with TSLP (10 ng/ml) or PBS for 24 h. Representative images of immunofluorescence staining with primary antibodies targeting (**A**) F4/80 and M1 macrophage marker NOS2, and (**B**) F4/80 and M2 macrophage marker CD206. Scale bars: 25 µm. **C** The ratio of M1 macrophages was quantified; n = 4 per group. **D** The ratio of M2 macrophage was quantified; n = 4 per group. **E** Flow cytometry of M1 macrophages in indicated cell groups and the quantification; n = 4 per group. **F** Flow cytometry of M2 macrophages in indicated cell groups and quantification; n = 4 per group; the data are presented as mean ± SD. Not significant is indicated as NS. *P < 0.05, **P < 0.01.

The importance of TSLP in asthma has been repeatedly documented. TSLP is mainly secreted by epithelial cells, airway smooth muscle cells, fibroblasts, mast cells, and macrophages/monocytes, and it is considered as an asthmatic inflammatory agonist [34, 35]. To further verify whether Gsdmd is involved in macrophage M2 polarization in an asthmatic inflammatory environment, we used TSLP to trigger inflammation from primary macrophages isolated from WT and *Gsdmd*^−/−^ mouse groups, and then analyzed M2 macrophage polarization by immunofluorescence staining and flow cytometry. The results showed that TSLP promoted M2 macrophage polarization from WT mice, but not from the *Gsdmd*^−/−^ mouse group (Fig. 7B, D). The M1 marker NOS2 showed no significant differences among the groups (Fig. 7A, C). Consistent result was also obtained by flow cytometry analysis (Fig. 7E, F). In summary, *Gsdmd* deficiency alleviates M2 polarization of macrophages in an asthmatic inflammatory environment.

### *Gsdmd* targets Notch signaling to regulate macrophage adhesion, and migration

**Fig. 8:**
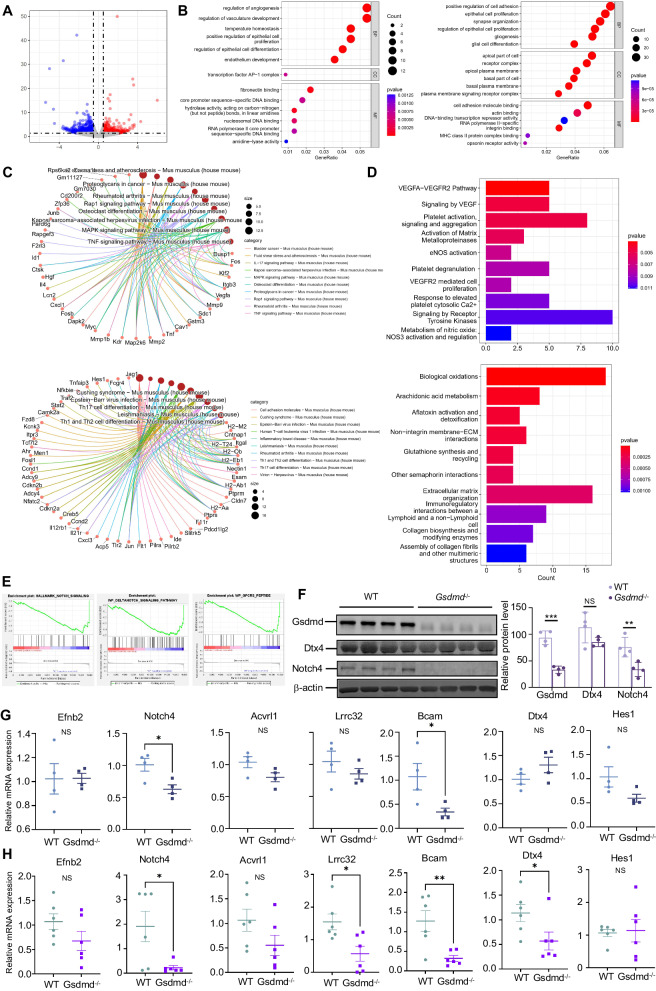
Gsdmd might target Notching signaling to regulate macrophage adhesion and migration. **A** Volcano plot of DEGs between macrophages isolated from WT and *Gsdmd*^−/−^ mice identified using edgeR algorithm, with the cut-off criterion *P* < 0.05 and |log2FC| ≥ 0.5. Blue dots: significantly downregulated genes; red dots: significantly upregulated genes. **B** GO analysis of DEGs (Left: upregulated genes; Right: downregulated gene) with three terms biological processes (BP), cellular components (CC), and molecular functions (MF) (*P* value < 0.05). **C** KEGG pathways enriched in the downregulated and upregulated risk-related DEGs (Upper: upregulated genes; Below: downregulated gene) (P value < 0.05). **D** Reactome pathway analysis of DEGs (Upper: upregulated genes, Below: downregulated genes) between macrophages obtained from WT and *Gsdmd*^*−/−*^ mice. **E** Gene set enrichment analysis (GSEA) of DEGs. **F** Expression of Notch4 and Dtx4 in the Notch signal pathway was examined in primary macrophages isolated from WT and *Gsdmd*^*−/−*^ *mice*. β-actin is the internal control. qPCR validation of key molecules in Notch signaling pathway in macrophages (**G**) and (**H**) lung tissues of wild type and *Gsdmd*^*−/−*^ mice. The data are presented as mean ± SD. Not significant is indicated as NS. *P < 0.05, **P < 0.01, ***P < 0.001.

To further explore the mechanism of Gsdmd in M2 macrophage polarization in asthma, we performed bulk RNA-seq on normal and *Gsdmd*^−*/−*^ macrophages isolated from bone marrow of WT and *Gsdmd*^*−/−*^ mouse model. A significant difference in DEGs was observed in macrophages isolated from WT and *Gsdmd*^*−/−*^ mice (Fig. 8A). GO analysis showed that *Gsdmd* knockout enriched gene sets related to regulation of angiogenesis, regulation of vasculature development, and regulation of epithelial cell proliferation and differentiation. Upregulated genes in *Gsdmd*^*−/−*^ macrophages were highly enriched in fibronectin binding, while downregulated genes were observed to be enriched in regulation of cell adhesion and MHC protein complex binding (Fig. 8B). These results suggested *Gsdmd* knockout may regulate inflammatory responses in macrophages. IL-17 induces the production of pro-inflammatory cytokines, chemokines, and growth factors in macrophages. KEGG analysis further indicated the regulation of Gsdmd in the inflammatory response as the IL-17 signaling pathway and the TNF signaling pathway were enriched in upregulated genes in *Gsdmd*^*−/−*^ macrophages (Fig. 8C). Consistent with GO analysis, KEGG analysis revealed that gene sets related to cell adhesion were enriched in downregulated genes in *Gsdmd*^*−/−*^ macrophages. Enrichment of the VEGFA-VEGFR2 and VESF pathways may once again reflect the role of Gsdmd in enhanced migration of macrophages (Fig. 8D). GSEA showed that the Notch signaling pathway as well as the GPCRS peptide pathway are downregulated in *Gsdmd*^*−/−*^ macrophages, suggesting that Gsdmd may have an impact on macrophage activation (Fig. 8E) [36, 37]. We subsequently verified the upregulation of Notch4 in macrophages isolated from WT mice as compared to those from *Gsdmd*^*−/−*^ mice at both mRNA (Fig. 8G) and protein level (Fig. 8F). Downregulated expression of key molecules in the Notch signaling pathway was detected in *Gsdmd*^*−/−*^ BMDMs compared with WT BMDMs. Likewise, the downregulation of the key molecules in the Notch signaling pathway was also validated in lung tissues of WT and *Gsdmd*^*−/−*^ mice (Fig. 8H). Collectively, Gsdmd might mainly get involved in positive regulation of macrophage adhesion and migration via targeting Notch signaling pathway.

## Discussion

The most traditionally accepted mechanism of asthma pathogenesis is airway inflammation and remodeling [38]. An emerging concept holds that airway inflammation launches the asthma pathogenesis progression, then promotes airway remodeling. These alterations are hypothesized to lead to airway hyperresponsiveness and progressive decline of lung function in asthmatic patients [39]. However, airway inflammation and remodeling are concurrent and interdependent events that promote and sustain each other rather than being sequential or consequential [40]. Thus, we explored the airway inflammation and remodeling simultaneously.

The human gasdermin family is important in pyroptosis, GSDM proteins (GSDMA/B/C/D) are expressed in epithelial cells in the maintenance of the epithelial cell barrier [41, 42]. Central to the inflammatory process of pyroptosis is the protein GSDMD [3]. Recent data have shown that GSDMD is expressed in various subsets of leukocytes and bronchial epithelial cells [43, 44], and in almost all organs and tissues, including the gut, heart, spleen, liver, and lung in human and mice [45, 46]. Meantime, a number of studies suggest that pyroptosis is involved in the pathogenesis of asthma. Studies have shown that allergen induced IL-1β and IL-18 release and barrier dysfunction via the NLRP3-caspase-1 in human bronchial epithelial cells [6, 47, 48]. Kinlin L. Chao et al found that the GSDMB polymorphism was a potential disease factor associated with asthma [8]. However, the role of the pyroptosis executor GSDMD in the pathophysiological process of asthma is little known. The main finding of our study was that N-GSDMD was upregulated in the airway epithelium in asthma patients, accompanied by higher expression of pyroptosis-associated inflammatory cytokines (IL-1β and IL-18). Here, we confirmed the clinical significance that GSDMD-induced pyroptosis plays an essential role in asthma patients.

In the present study, we further investigated GSDMD-induced pyroptosis and its role in the immune response of airway inflammation and remodeling in asthma model. Exogenous and endogenous damage are recognized by the intracellular sensor proteins such as NLRP1, NLRC4, NLRP3, AIM2, and Pyrin, leading to the action of caspase-1 in the canonical pathway and caspase-11 in the non-canonical pathway in mice [49, 50]. Activated caspase-1 and caspase-11 cleave GSDMD, and then the GSDMD N-terminal domain forms pores on cell membrane, leading to the generation of the cleaved pro-inflammatory cytokines IL-1β and IL-18. Consequently, matured IL-1β and IL-18 release from the pores on the cell membrane. [51, 52]. Consistent with other reports, airway inflammation and AHR were significantly increased in OVA-sensitized WT mice [53]. Conversely, Gsdmd-deficient mice exhibited reduced airway inflammation and AHR upon OVA challenge. Our data demonstrated that the pathophysiology of asthma might be dependent on GSDMD-induced pyroptosis.

It is well known that the Th1/Th2 balance and M2 macrophage polarization play central roles in the pathogenesis of asthma, but the Th1/Th2 balance or M2 macrophage polarization alone cannot explain the mechanism of asthma. A number of studies have shown that allergen-induced airway inflammation in mice is mediated by immunoregulation of Th17/Treg responses, characterized by increased Th17 cells and the decreased Treg cells [54, 55]. Based on the previous reports, we detected the Th17 and Treg cell related inflammatory cytokines (IL-17A and IL-10) in the serum of asthma patients. A noticeably high level of IL17A, and a reduction in IL-10 were observed. Here, we also obtained consistent results that the levels of serum IL-17A and Th17 cell proportions in the spleen were significantly higher in WT asthmatic mice than that in the control group, while the levels of serum IL-10 and Treg cell proportion in spleen were markedly lower in WT asthmatic mice than in control WT mice. However, *Gsdmd* knockout reversed the Th17/Treg imbalance led by OVA induction in the asthmatic mice. Meanwhile, we also found that *Gsdmd* knockout alleviated the M2 macrophage polarization in the lung tissues and TSLP-stimulated macrophages. Moreover, we found that GSDMD may target Notch signaling to regulate macrophage adhesion and migration by bulk RNA-seq analysis. The results were then confirmed in cultured primary macrophage obtained from WT and *Gsdmd*^*−/−*^ mice. Therefore, we confirmed that GSDMD-induced pyroptosis is involved in the pathogenesis of asthma by regulating both Th17/Tregs imbalance and M2 macrophage polarization. In addition, we showed that increased collagen deposition, and mucus production in lungs of OVA-sensitized WT mice were rescued in the *Gsdmd*^*−/−*^ mice. These findings demonstrated that GSDMD-induced pyroptosis in airway inflammation and remodeling by the immunoregulation of Th17/Treg and M2 macrophage polarization is worthy of future investigation.

**Fig. 9:**
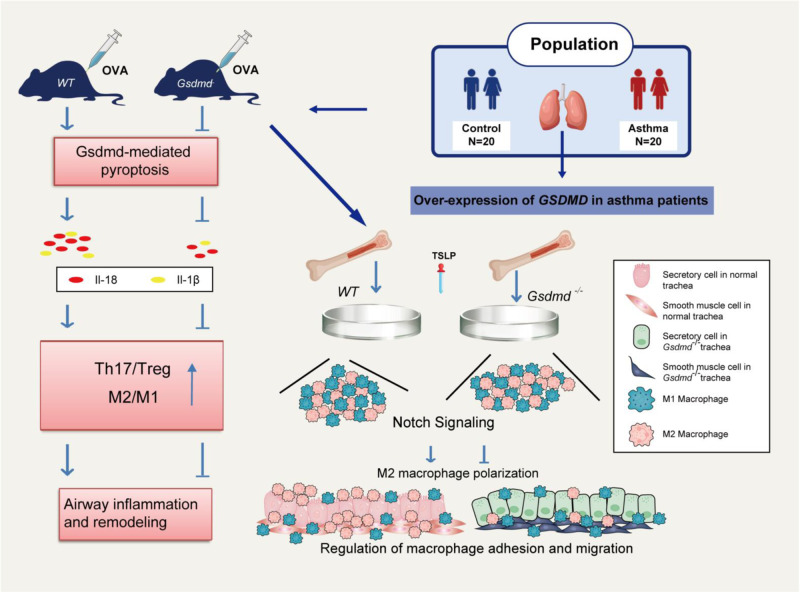
Schematic diagram shows the function and pathway of GSDMD in asthmatic airway inflammation and remodeling. The expression of N-GSDMD and pyroptosis-related cytokines is significantly increased in the airway epithelium and serum from asthma patients. Pyroptosis is enhanced in an OVA-induced asthma mouse model. *Gsdmd* deficiency reduces pyroptosis in the asthmatic mice. *Gsdmd* deficiency in mice significantly attenuates Th17 and Th2 inflammatory responses and M2 macrophage polarization, which contribute to airway inflammation and remodeling. Notch signaling may regulate M2 macrophage polarization upon *Gsdmd* deletion. The data demonstrate that GSDMD promotes allergic inflammation and tissue remodeling in asthma.

We acknowledged several limitations of our study. First, we only detected the N-GSDMD expression in the airway epithelium accompany with IL-18 and IL-1β in serum. Second, GSDMD is extensively expressed in multiple cell types in the lung tissue. However, the decisive sources of GSDMD in the pathogenesis of asthma have not been determined yet. The role of GSDMD plays on other cell types still requires further investigation. Moreover, the biopsy sample size was relatively small so we did not explore the correlation between pyroptosis level and severity of the illness. In the future, we will further investigate the other potential mechanism in the regulation of asthma. Taken together, our results suggest that GSDMD-induced pyroptosis is upregulated in asthma patients. Furthermore, GSDMD is involved in asthmatic airway remodeling and inflammation by the immunoregulation of Th17/Treg and M2 macrophage polarization targeting Notch signaling pathway (Fig. 9). These findings indicate that GSDMD may be a novel target for the treatment of inflammation and airway remodeling in asthma.

### Supplementary information

Supplementary Fig. 1

Supplementary Fig. 1 Legend

The original bands of the western blot

## Data Availability

The RNA-seq expression data of mouse BMDMs has been deposited in the Gene Expression Omnibus (GEO) under accession number GSE249948. This paper does not report original code. Any additional information required to reanalyze the data reported in this paper is available from the lead contact upon request.
