# Supplementary figures and images for: Gasdermin D silencing alleviates airway inflammation and remodeling in an ovalbumin-induced asthmatic mouse model
Source: Cell Death Dis. 2024 Jun 7;15(6):400. doi: 10.1038/s41419-024-06777-5 (PMC11161474; doi:10.1038/s41419-024-06777-5)

A

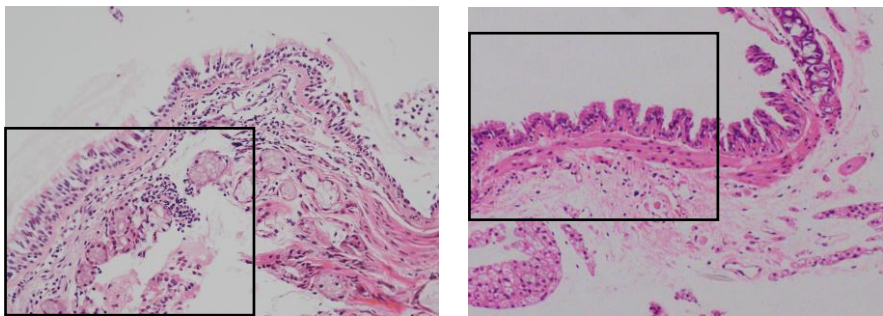

B

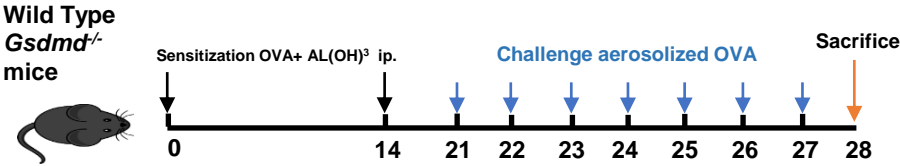

Supplement: Supplementary file 1 — Supplementary Fig. 1 [file 41419_2024_6777_MOESM1_ESM.pdf]
