# Supplementary material for: Gasdermin D silencing alleviates airway inflammation and remodeling in an ovalbumin-induced asthmatic mouse model
Source: Cell Death Dis. 2024 Jun 7;15(6):400. doi: 10.1038/s41419-024-06777-5 (PMC11161474; doi:10.1038/s41419-024-06777-5)
Supplement: Supplementary file 2 — Supplementary Fig. 1 Legend [file 41419_2024_6777_MOESM2_ESM.docx]

**Supplementary Fig. legend**

**Supplementary Fig. 1 A** The morphological changes of airway epithelium in asthma patients and control group were observed by HE staining. **B** Sensitization and challenge protocol for construction of OVA-induced asthma in WT and *Gsdmd^-/-^* mice.
