# Supplementary material for: Gasdermin D silencing alleviates airway inflammation and remodeling in an ovalbumin-induced asthmatic mouse model
Source: Cell Death Dis. 2024 Jun 7;15(6):400. doi: 10.1038/s41419-024-06777-5 (PMC11161474; doi:10.1038/s41419-024-06777-5)

Fig 2E

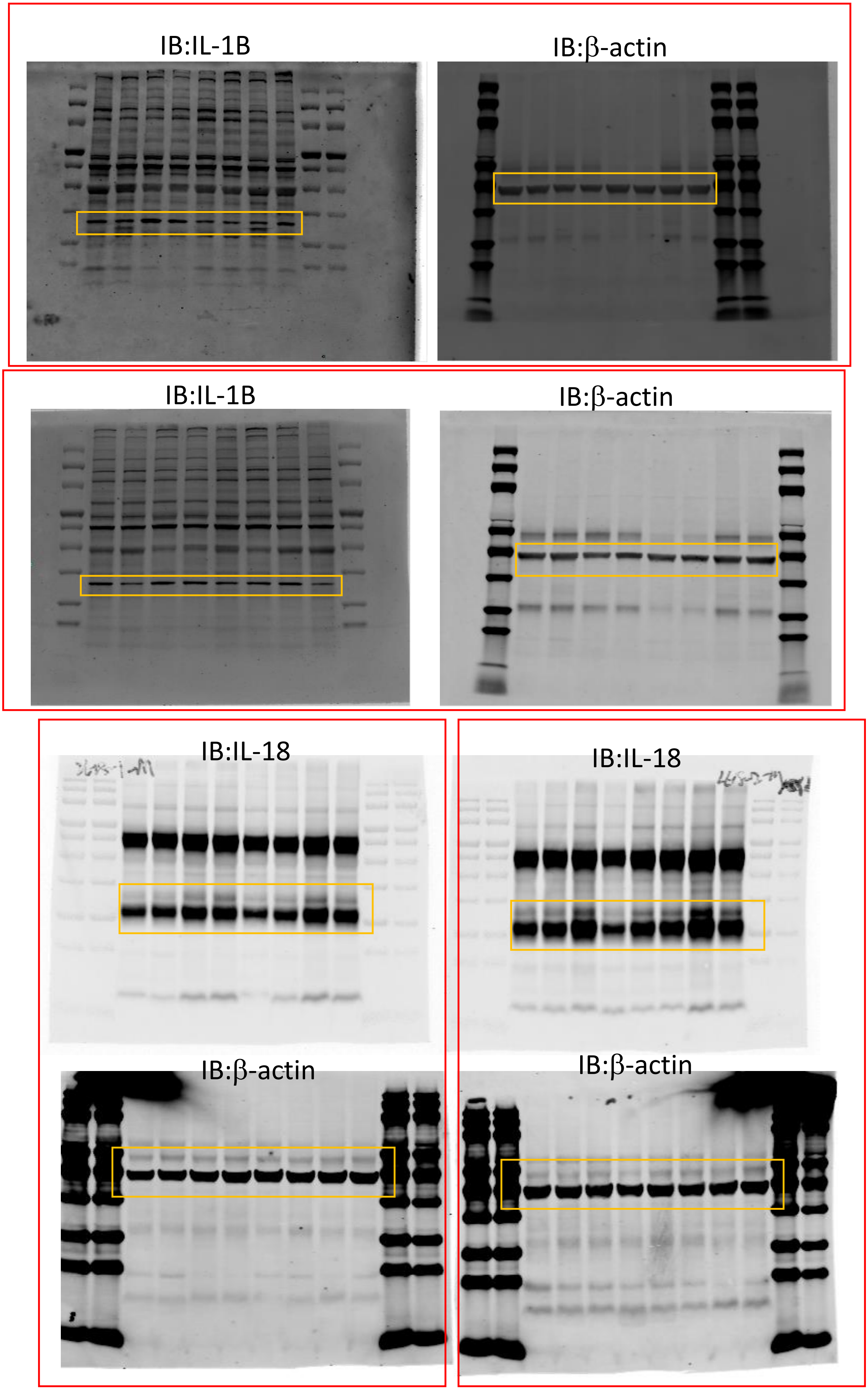
IB:IL-1B
IB:β-actin
IB:IL-1B
IB:β-actin
IB:IL-18
IB:IL-18
IB:β-actin
IB:β-actin

Fig 2E

IB: Gsdmd

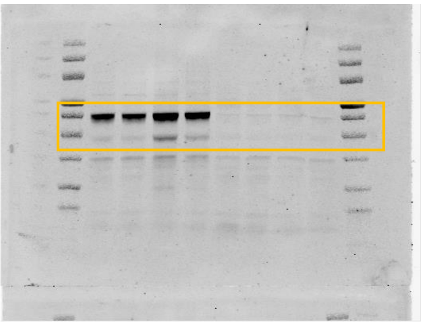

IB:β-actin

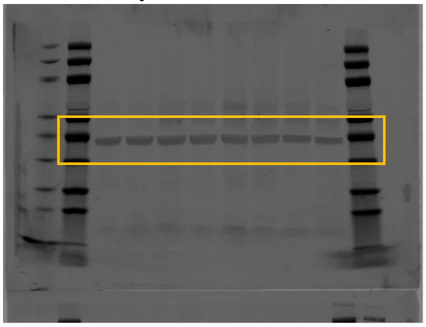

IB: Gsdmd

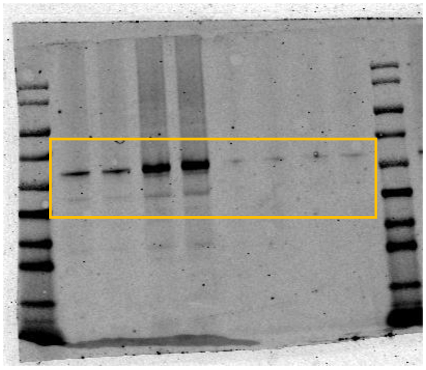

IB: Caspase-11

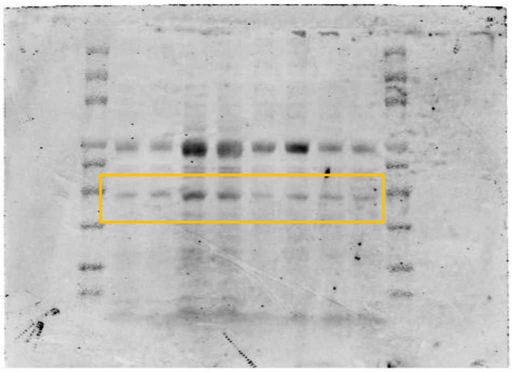

IB: Caspase-1

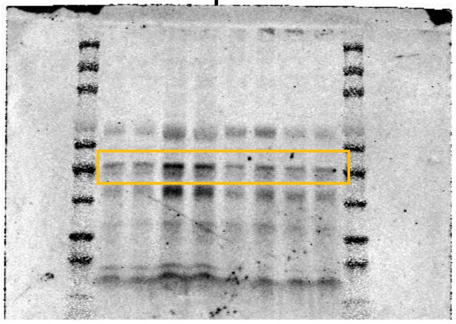

IB: β-actin

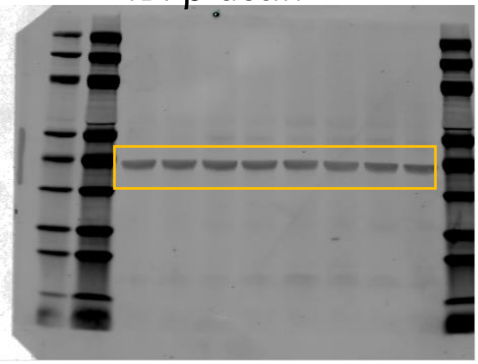

IB: Caspase-1

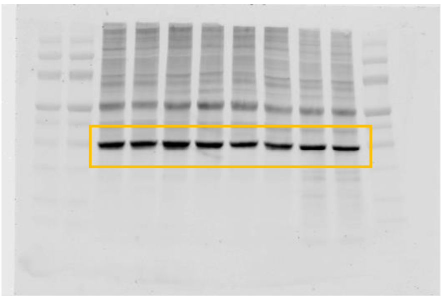

IB: β-actin

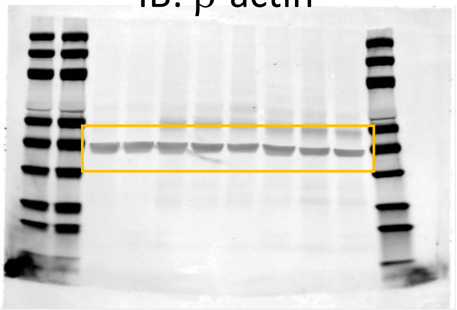

Fig 2E

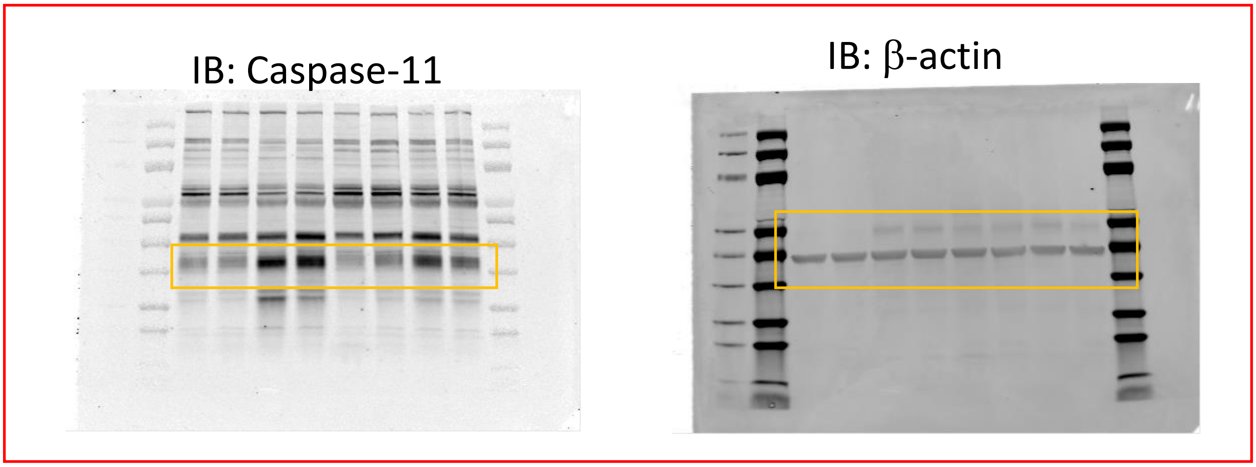

Fig 8F

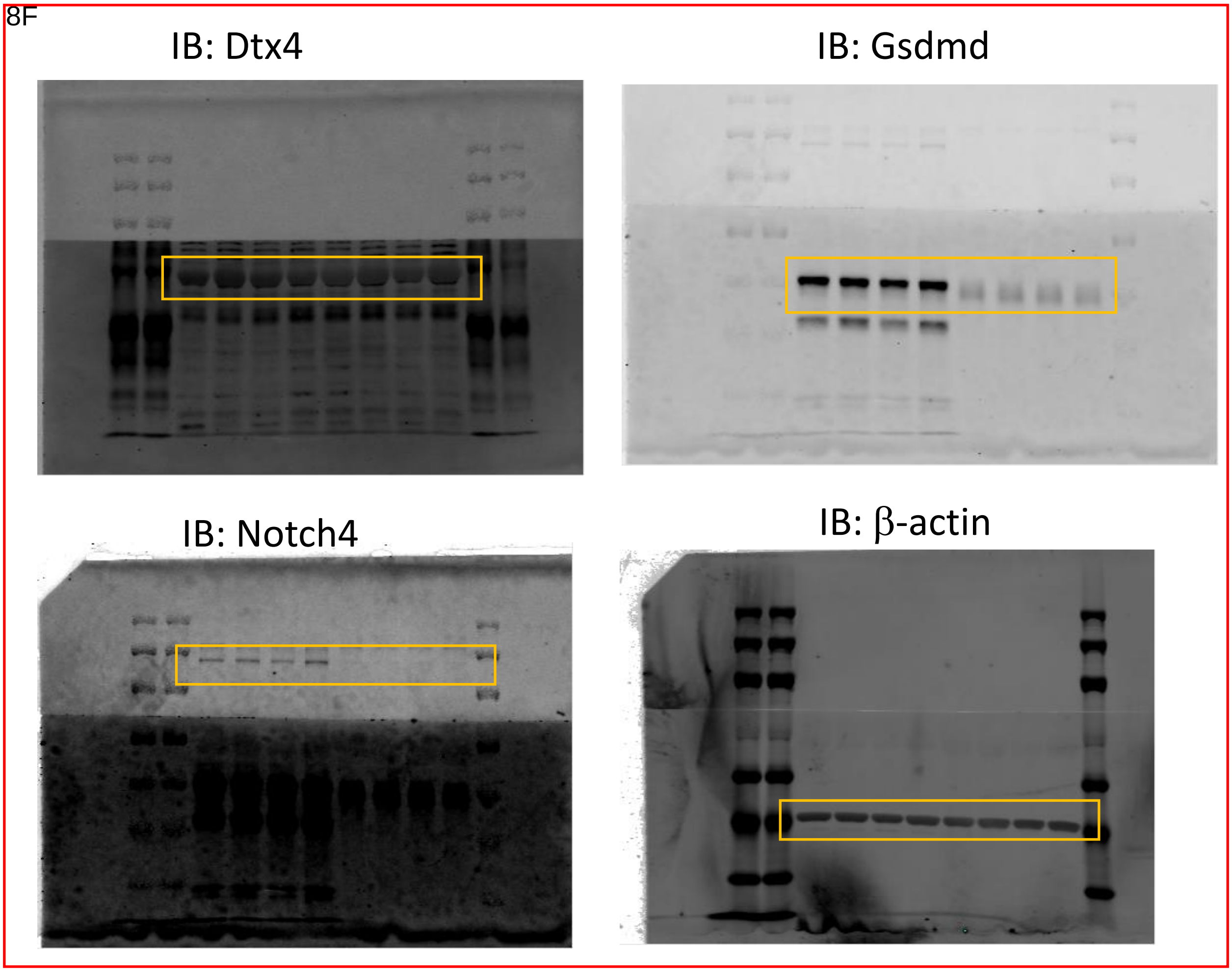

Supplement: Supplementary file 3 — The original bands of the western blot [file 41419_2024_6777_MOESM3_ESM.pdf]
